# House-hold contact tuberculosis screening adherence and associated factors among tuberculosis patients attending at health facilities in Gondar town, northwest, Ethiopia

**DOI:** 10.1186/s12879-019-4695-7

**Published:** 2019-12-18

**Authors:** Dessie Alemnew Shiferaw, Habtamu Sewunet Mekonnen, Addisu Taye Abate

**Affiliations:** 1 Department of Nursing, Debre-Tabor Health Science College, Debre-Tabor, Ethiopia; 2 Department of Medical Nursing, School of Nursing, College of Medicine and Health Sciences, University of Gondar, Gondar, Ethiopia

**Keywords:** Contact screening, Household, Adherence, Tuberculosis, Ethiopia

## Abstract

**Background:**

Contacting patients with tuberculosis have a substantial risk of developing the disease. Household contact screening has recently been recommended as a strategy to enhance case detection in high-burden countries. But there is no enough information in Gondar town regarding household contact screening practice among TB patients.

**Methods:**

An institution-based cross-sectional study was conducted from March 1 to 30, 2019 on 404 tuberculosis patients attending at health facilities in Gondar Town. Epi-Info version 7 for data entry and SPSS version 20 for data analysis were used. Descriptive statistics were carried out to illustrate the means, standard deviations, and frequencies. Bivariable and multivariable logistic regression analyses were used to identify significantly associated variables with the dependent variable.

**Results:**

From 412 study populations, 404 were completed the study with 98.06% response rate. The overall household contact TB screening adherence was 47.5% (95% CI: 43.1, 52.5). In the multivariable analysis, having certificate and above educational level (AOR = 2.83, 95% CI:1.40,5.67), having sufficient knowledge about TB (AOR = 8.26, 95% CI:4.34,15.71), being satisfied with health care service (AOR = 3.26, 95% CI:1.58,6.76), health education given by health care workers (AOR = 2.60, 95% CI:1.54,4.40),and having HIV/AIDS co-infection (AOR = 3.54, 95% CI:1.70,7.39), were factors associated with household contact TB screening adherence.

**Conclusion:**

Compared to other previous studies, the current finding was high but it was low as compared with WHO and Ethiopian Ministry of Health recommendations (all persons having TB contact should be screened). Educational status, knowledge on TB, satisfaction with delivered health care service, health education given by HCWs about TB and HIV/AIDS co-infection were factors associated with household contact TB screening practice. Thus, strengthening household TB contact screening and educational programs regarding the risk of getting TB infection from household contacts is crucial.

## Background

Tuberculosis is an airborne contagious disease mainly caused by *Mycobacterium tuberculosis*. When TB-infected individuals cough, sneeze and shout; Mycobacterium in the suspended air can enter the respiratory system of a nearby healthy person. There are two types of TB named pulmonary and extrapulmonary. Pulmonary tuberculosis occurs when *Mycobacterium tuberculosis* primarily attacks the lungs. However, it can spread to other organs causing extrapulmonary TB [1–3].

Globally, there were an estimated 10.0 million new cases of TB disease (also known as active TB) in 2017. Even though, tuberculosis is an easily preventable and treatable disease, it remains a major cause of morbidity and mortality in many countries and an important public health problem of the world. TB is one of the top 10 causes of death and it is responsible for 1,600,000 TB related global deaths in 2017 [3]. A study conducted in New Delhi revealed that contacts of pulmonary positive TB patients have significantly higher infection rates as compared to contacts of pulmonary negative TB patients [4].

Ethiopia is one of the 30 high-TB and multidrug resistance TB-burden countries. According to the 2017 World Health Organization (WHO) report, Ethiopia is the 10th from 30th high TB and HIV burden countries with an incidence of 177 per 100,000 among all forms of TB. Thus, the government of Ethiopia has given due attention for the prevention and control of TB and it is the priority health program of the country’s Health Sector Development Program (HSDP) but is not fast enough to achieve 2020 stop TB strategy [5].

TB contact patients are the high-risk group to develop TB, especially children less than 5 years and people living with HIV are at higher risk [6–8]. Household contacts with active TB cases are at high risk of getting TB disease [9, 10]. Major challenges to the successful control of TB includes; timely diagnosis and adequate treatment of active TB (the index case), contact investigation (CI) or screening of persons in close contact with the index case, and treatment of latent tuberculosis infection (LTBI) to prevent its progression to active disease [10]. These three core elements are the basis of TB control [11]. Contact investigation, as one of the core element, is an important policy of TB control and play an important role to decrease the incidence [12].

The magnitude of TB screening adherence in Thailand, Southeast Nigeria, Gambo rural hospital, and urban districts of Amhara region in Ethiopia was 52, 23.6, 55.7, 33.7% respectively [13–16]. Regarding the associated factors, marital status, religion, family income, relationship with contact, knowledge, sharing a bedroom and type of TB were significantly associated factors with TB screening adherence. Besides distance from health-care facilities, lack of transport, direct and indirect costs of health care all present barriers to access household contact screening [15–17].

Screening of specific risk groups like household contacts, have been part of the Stop TB Strategy since its launch in late 2012 [18, 19]. Despite, household contact TB screening adherence is an important component to achieve the strategy of stop TB, there is no study conducted in Gondar town health facilities regarding household contact screening adherence on TB patients. Few studies conducted in Ethiopia were on the magnitude of household contact screening practice and othrs were limited in numbers of factors that influence household contact screening adherence. Therefore, the aim of this study was to assess the magnitude of household contact screening adherence and associated factors among TB patients attending at health facilities in Gondar Town.

## Methods

### Study design and period

An institution-based cross-sectional study was conducted from March 1 to 30, 2019.

### Study area

The study was conducted in Gondar town, Northwest Ethiopia. Gondar town is one of the historical towns in Ethiopia. It is found about 737-km away from Addis Ababa. In the town, there are one governmental specialized Hospital, eight governmental health centers, one private Hospital, and one private clinic, which delivers anti TB treatment and screening service. Currently, about 483 TB patients attend in these health facilities, of those 412 were adults and had household contact history.

### Source population

The source population constitutes all adult patients who have anti-TB treatment follow up at health institutions in Gondar town and had household contact.

### Study population

All adult patients with TB who had household contact, anti-TB treatment follow up at health facilities in Gondar town and found during the specified study period.

### Inclusion criteria

All adult patients with TB who had household contacts and anti-TB treatment follow up in Gondar town health facilities were included.

### Exclusion criteria

Adult patients with TB who had household contacts and were critically ill and unable to communicate, patients start treatment at the day of data collection.

### Sample size determination and sampling procedure

The sample size was determined using a single population proportion formula, taking 33.7% magnitude of household contact screening practice of a study conducted in Amhara Region, Northwest Ethiopia [16] with the following assumptions: 95% CI and 5% margin of error and by adding 10% none response rate. The total sample size was 379. The study populations were 412 closes to the calculated sample. So, all the study populations were included in the study.

### Operational definition

#### Household contact

A person who shared the same enclosed living space for one or more nights with the index case during the 3 months before the commencement of the current treatment episode [16, 20, 21].

#### Household contact TB screening adherence

If the patient brought at least one household contact for TB screening and otherwise not-adherent [16].

#### Patient with sufficient knowledge on TB

A patient who answered greater than or equal to 80% of the given TB related knowledge questions [16].

#### HIV AIDS co-infection

Presence of confirmed HIV/AIDS along with TB [22].

#### Waiting time

Time taken to get service after the arrival of health facilities [13].

#### Patients who were satisfied by the services delivered at health facilities

Those respondents who scored points ≥ 75% of the given satisfaction related questions otherwise unsatisfied [23, 24].

#### Index case

Refers to TB patient who is initially diagnosed with infectious TB, and around him, contact investigation for a potentially exposed individual is indicated [21].

### Data collection tool and procedures

A pre-tested and structured interviewer-administered questionnaire was used. First the questionnaire was prepared in the English language and it was translated to the local language (Amharic); then to ensure its consistency it was re-translated to English language by language expert. The questionnaire had five sections; Socio-demographic characteristics of the participants, practice, personal and behavioral factors of the participants, health care system-related factors, disease and treatment conditions associated with the practice. Participants medical documents were reviewed to collect information about the type of TB, date of diagnosis and HIV/AIDS infection status, and to confirm the verbal reports of contact schreening status. Participants were interviewed after briefly explained the study purpose and getting consent from each individual patient.

### Data quality assurance

To test the fitness of the questionnaire for the study settings, the questionnaire was pretested taking 5% of the sample (20 TB patients) in Addis Zemen Hospital and health center prior to the actual study. Two days training was given for data collectors and supervisors about the data collection tool and data collection procedures. The data were checked for its completeness and accuracy.

### Data processing and analysis

After data collection, a questionnaire was checked for completeness and consistency. The data were entered in to Epi-Info version 7 and then export to SPSS version 20 software for analysis. Descriptive statistics were carried out to illustrate the means, standard deviations, and frequencies. Tables and figures were used to display the findings. Binary logistic regression analysis was done to identify variables having a significant association with the dependent variable. Then control the effect of confounding, all independent variables with a *p*-value less than 0.2 in the bivariate analysis were again entered to multivariable logistic regression. Finally, variables with *P*-value less than 0.05 considered as significantly associated factors. Hosmer and Lemeshow goodness of fit test was used for model fitness.

## Results

### Socio-demographic characteristics of respondents

From 412 study participants, 404 were complete the study with 98.06% response rate. Among the participants, 144 (35.6%) were in the age group of 20–29 years, with the mean age of 35.75 years (±14.28 standard deviation). Two hundred twenty-one (54.7%) males. Two hundred (49.5%) of the participants were married and majority 386 (95.5%) were Amhara by Ethnicity. One hundred thirty-two (32.7%) were merchant by occupation122(30.2%) participants had a primary school educational status. From all study participants, 283 (70%) had > = 1001 ETB average monthly income. About 210 (52%) of respondents had contact relationship with their son/daughter (Table 1).

**Table 1:** Socio-demographic characteristics of Tuberculosis patients attending at health facilities in Gondar Town, Northwest Ethiopia, March 2019 (*n* = 404)

Variables	Frequency (n)	Percent (%)
Age		
< 20	17	4.2
20–29	144	35. 6
30–39	108	26.7
40–49	61	15.1
> =50	74	18.3
Sex		
Male	221	54.7
Female	183	45.3
Marital status		
Single	147	36.4
Married	200	49.5
Divorced	43	10.6
Widowed	14	3.5
Ethnicity		
Amhara	395	97.77
Oromo	9	2.2
Educational status		
No formal education	110	27.2
Primary	122	30.2
Secondary	83	20.5
Certificate and above	89	22.0
Occupation		
Governmental Employed	68	16.8
Farmer	56	13.9
Merchant	132	32.7
Student	69	17.1
Housewife	59	14.6
Daily laborer	20	5
Family monthly income(Ethiopian Birr)		
< =300	27	6.7
301–600	38	9.4
601–1000	56	13.9
> =1001	183	70
Relationship of household contacts with respondents		
Spouse	184	45.5
Father	56	13.9
Mother	96	23.8
Sister/brothers	32	7.9
Relatives	52	12.9
Son/daughter	210	52.0
Friends	97	24

### Personal, health care system and disease-related characteristics

From a total of 404 study participants, 285 (70.5%) had sufficient knowledge about TB. Concerning to causes of TB 209(51.7%) believed that bacteria cause it and 348(86.1%) of them perceived as TB is transmitted through infectious droplets released from TB patients. Majority, 332(82.2%) of the participants were satisfied with TB clinic service. Two hundred (49.5%) and 152(37.6%) were patients with drug-sensitive Pulmonary Tuberculosis (PTB) and drug-sensitive extrapulmonary tuberculosis (EPTB) respectively. Two hundred forty (59.4%) participants were on a continuation phase of anti TB treatment. Regarding HIV/AIDS co-infection status of the participants, 60(14.9%) of were HIV infected along with TB. (Table 2).

**Table 2:** Personal, Health care system-related characteristics of respondents, health facilities in Gondar Town, Northwest Ethiopia, March 2019(*n* = 404)

Variables	Frequency(n)	Percent (%)
Knowledge of TB		
Sufficient	285	70.5
Insufficient	119	29.5
Patient satisfaction by service delivered at health facilities		
Satisfied	335	82.9
Unsatisfied	69	17.1
Type of health facility		
Governmental hospital	130	32.2
Governmental health center	250	61.9
Private hospital	11	2.7
Private clinic	13	3.2
Mode of transportation		
On foot	222	55.0
Public transport	182	45.0
Health education about TB		
Yes	253	62.6
No	151	37.4
Waiting time at TB clinic		
< 60 min	332	82.2
> =60 min	72	17.8
Type of TB		
Drug sensitive PTB	200	49.5
Drug sensitive EPTB	152	37.6
MDR TB	52	12.9
Phase of TB treatment		
Intensive phase	164	40.6
Continuation phase	240	59.4
HIV co -infection		
Yes	60	14.9
No	344	85.1`

### Household contact TB screening adherence

In this study, the overall prevalence of household contact screening adherence was 47.5% (95% CI: 43.1, 52.5). The mean number of household contacts of participants was 3.56 with a Standard Deviation of ±2.09 and the mean number of household contacts brought for screening purpose was 2.16 with Standard Deviation of ±1.24.

### Factors associated with household contact TB screening adherence

In the bivariable logistic regression analysis, household contact-screening adherence was significantly associated with educational status, knowledge about TB, health education given by health care workers about TB, patient satisfaction by health facilities service, type of tuberculosis, and HIV/AIDS co-infection.

However, in multivariable logistic regression, educational status, knowledge about TB, health education is given by health care workers about TB, patient satisfaction by health facilities service, and HIV/AIDS co-infection were significantly associated with household contact screening practice. Participants who had a certificate and above educational level were nearly three times more likely to practice household contact screening as compared with who were not formally educated (AOR = 2.82, 95% CI: 1.40, 5.67). Patients with sufficient knowledge on TB were eight times (AOR = 8.26, 95%CI: 4.34, 15.72) more likely to practice household contact screening as compared with who had insufficient knowledge. Participants who were satisfied by delivered health care services were three times (AOR = 3.26, 95% CI: 1.58, 6.76) more likely to practice household contact screening as compared to patients who were not satisfied.

Patients who took health education from HCWs were nearly three times (AOR = 2.60, 95% CI: 1.54, 4.40) more likely to practice household contact screening as compared to patients who did not take health education. Patients who had HIV/AIDS co-infection were 3.5 times (AOR = 3.54, 95% CI: 1.70, 7.39) more likely to practice household contact screening as compared to patients who had no HIV/AIDS co-infection (Table 3).

**Table 3:** Bivariable and Multivariable logistic regression analysis of factors for household contact screening adherence, health facilities in Gondar Town, North West Ethiopia, March 2019 (*n* = 404)

Variable	HHCS adherence		COR (95% CI)	AOR (95% CI)
	Yes	No		
Educational status				
No formal education	46	64	1	1
Primary education	54	68	1.11 (0.66,1.86)	1.03(0.54,1.93)
Secondary education	37	46	1.12 (0.63,1.99)	1.09(0.54,2.20)
Certificate and above	55	34	2.25(1.27,3.99)	2.82(1.40,5.67)**
Marital status				
Single	67	80	1	1
Married	103	97	0.79(0.52, 1.21)	0.88(0.6,2.01)
Divorced	15	28	1.56(0.77,3.17)	1.70(0.90,4.11)
Widowed	7	7	0.84(0.28,2.51)	1.47(0.36, 3.22)
Family monthly income (Ethiopian Birr)				
< =300	8	19	2.33(0.99, 5.49)	2.60(0.89, 6.54)
301–600	16	22	1.35(0.68, 2.67)	2.92(0.78, 3.70)
601–1000	28	28	0.98(0.55, 1.74)	0.83(0.66, 2.03)
> =1001	140	143	1	1
Type of health facility				
Governmental hospital	63	67	1	1
Governmental health center	118	132	1.05(0.69,1.61)	1.45(0.77,1.82)
Private hospital	5	6	1.13(0.33, 3.88)	1.92(0.46,4.14)
Private clinic	6	7	1.10(0.35, 3.44)	1.32(0.60, 3.99)
Mode of transportation				
On foot	97	129	1.65(0.98,2.45)	1.74(0.85,3.33)
Public transport	99	3	1	1
Knowledge on TB				
Sufficient	176	109	10.39(5.83,18.53)	8.26(4.34,15.72)**
Insufficient	16	103	1	1
Patient satisfaction by health care service				
Yes	178	157	4.45(2.39,8.32)	3.26(1.58,6.76)**
No	14	55	1	1
Health education about TB by HCW				
Yes	155	98	4.87(3.11,7.63)	2.60(1.54,4.40)**
No	37	114	1	1
Type of Tuberculosis				
Drug-sensitive PTB	93	107	1	1
Drug-sensitive EPTB	65	87	0.86(0.56,1.31)	1.09(0.65,1.83)
MDR TB	34	18	2.17(1.15,4.10)	1.54(0.72,3.31)
Phase of TB treatment				
Intensive phase	69	95	1.45(0.97, 2.16)	1.67(0.70(2.63)
Continuation phase	123	117	1	1
HIV/AIDS co infection				
Yes	40	20	2.53(1.42,4.50)	3.54(1.70,7.39)**
No	152	192	1	1

Key: HHCS-household contact screening, ** = significant with *p* < 0.005

## Discussion

The overall household contact screening practice was 47.5% (95% CI:42.2,52.2). The finding was in-line with the practice level reported in Bangkok, Thailand (52%) [13]. This finding was exceeded the findings of Enugu and Ebonyi States, Southeast Nigeria and in Amhara region (23.6, 33.7%,) respectively [14, 16]. The possible explanations could be differences in socio-cultural status of the index cases and health policy and health care system in the countries as well in study settings. The study in Southeast Nigeria was done only in governmental facilities but the current study was done in both governmental and private facilities. Besides, it could be due to time period in which studies in South Nigeria and Amhara region were done before 5 years In contrast, it was lower compared to a study done from Gambo Rural Hospital, Ethiopia which showed that 55.7% of the index cases brought at least one household contact for screening [15]. This difference might be due to the study design and source of information differences for the studies. The study done in Gambo rural Hospital was done by logbook review/taking secondary data and it was done only in governmental hospital but the current study is the primary data and it was done in both governmental and private Hospitals and health centers.

Educational status of respondents was significantly associated with household contact screening practice. Tuberculosis patients who had a certificate and above level of education were nearly three times more likely to practice household contact screening as compared to who had no formal education. This might be due to educated individuals read and listen to routine distributed information on TB through different media and easily understand risks for TB transmission and the benefits of contact investigation [16].

In this study, Knowledge of participants about TB and its’ treatment were found to be the factor for household contact tuberculosis screening practice. Thus, patients who had sufficient knowledge about tuberculosis were eight times more likely to practice household contact screening as compared to who had insufficient knowledge about TB. The Comparable finding was obtained from a study conducted in Amhara region, Northern Ethiopia two times more likely [16]. And in Bangkok, Thailand five times more likely to practice household contact tuberculosis screening as compared with who had no sufficient knowledge [13]. It was also consistent within Enugu, Southeast Nigeria revealed that knowledge about contact tracing has significant association on household contact screening practice [25].

Participants who had sufficient knowledge about TB had a good understanding of the risks of household contact for tuberculosis and the benefit of bringing household contact to TB screening among tuberculosis patients might contribute to their practice. In this study, patient satisfaction by health facilities service, plays a crucial role to practice household contact screening. Patients who were satisfied by health care service were three times more likely to practice household contact screening as compared to patients who were not satisfied. This might be because health care service satisfaction at health facilities might increase healthcare-seeking behavior among TB patients.

Health education given by HCWs was significantly associated with household contact TB screening adherence, as patients who took health education, were three times more likely to adhere to household contact screening. A similar study conducted in Vietnam showed that majority of the patients went for screening as a result of instructions provided by the health education [26]. This finding was also shown in another study done in Southeast Nigeria [25]. This finding was also consistent with a study conducted in Northern Ethiopia. It revealed that awareness creation about contact tracing has a significant association with household contact screening adherence [16]. This might be due to health education about TB at health facilities that focus on signs and symptoms of TB, the advantage of early screening and TB infection prevention techniques so those awareness creation activities might increase household contact TB screening practice among TB patients.

In this study, the household contact TB screening adherence among HIV infected participants was nearly four times more likely to practice household contact TB screening as compared with non-infected participants. This might be since HIV infected patients have regular visits to a health facility for antiretroviral therapy follow up. This implies that when patients came to health facilities frequently, their awareness about household contact screening practice also increased.

### Limitations of the study

In this study there may be social desirability bias from participants towards which they assumed good response even though; we tried to overcome by select data collectors from health professionals working outside the TB clinic. In addition, this study did not included the determinant factors of household TB contact members.

## Conclusion

The overall household contact TB screening practice was 47.5% (95% CI: 43.1, 52.5). As compared to other previous studies findings, household contact screening practice among tuberculosis patients attending in Gondar Town health facilities was high but it was low as compared with WHO and Ethiopian Ministry of Health recommendations. Educational status, knowledge on TB, satisfaction with delivered health care service, health education given by HCWs about TB and HIV/AIDS co-infection were factors associated with household contact TB screening practice. Therefore, strengthening household TB contact screening and educational programs regarding the risk of getting TB infection from household contacts is mandatory.

## Data Availability

The raw data would not be provided for the reason of protecting patients’ confidentiality. But the summary data are available in the main document.

## Abbreviations

AOR: Adjusted Odds Ratio
CI: Confidence Interval
COR: Crude Odds Ratio
EPT: Extrapulmonary Tuberculosis
HCW: Health Care Worker
HIV: Human Immune Deficiency Virus
HIV/AIDS: Human Immune Deficiency Virus/ Acquired Immuno-deficiency syndrome
HSDP: Health Sector Development Program
MDR-TB: Multidrug Resistance Tuberculosis
OR: Odds Ratio
PTB: Pulmonary Tuberculosis
SD: Standard Deviation
SPSS: Statistical Package for Social Sciences
TB: Tuberculosis
WHO: World Health Organization
